# Forest carbon in North America: annual storage and emissions from British Columbia’s harvest, 1965–2065

**DOI:** 10.1186/1750-0680-7-8

**Published:** 2012-07-24

**Authors:** Caren C Dymond

**Affiliations:** 1 Ministry of Forests, Lands and Natural Resource Operations, Government of British Columbia, PO Box 9515, Stn. Provincial Government, Victoria, BC V8W 9C2, Canada

**Keywords:** Forest products, C-accounting, Life-cycle analysis, Building science, Landfill emissions

## Abstract

**Background:**

The default international accounting rules estimate the carbon emissions from forest products by assuming all harvest is immediately emitted to the atmosphere. This makes it difficult to assess the greenhouse gas (GHG) consequences of different forest management or manufacturing activities that maintain the storage of carbon. The Intergovernmental Panel on Climate Change (IPCC) addresses this issue by allowing other accounting methods. The objective of this paper is to provide a new model for estimating annual stock changes of carbon in harvested wood products (HWP).

**Results:**

The model, British Columbia Harvested Wood Products version 1 (BC-HWPv1), estimates carbon stocks and fluxes for wood harvested in BC from 1965 to 2065, based on new parameters on local manufacturing, updated and new information for North America on consumption and disposal of wood and paper products, and updated parameters on methane management at landfills in the USA. Based on model results, reporting on emissions as they occur would substantially lower BC’s greenhouse gas inventory in 2010 from 48 Mt CO_2_ to 26 Mt CO_2_ because of the long-term forest carbon storage in-use and in the non-degradable material in landfills. In addition, if offset projects created under BC’s protocol reported 100 year cumulative emissions using the BC-HWPv1 the emissions would be lower by about 11%.

**Conclusions:**

This research showed that the IPCC default methods overestimate the emissions North America wood products. Future IPCC GHG accounting methods could include a lower emissions factor (e.g. 0.52) multiplied by the annual harvest, rather than the current multiplier of 1.0. The simulations demonstrated that the primary opportunities for climate change mitigation are in shifting from burning mill waste to using the wood for longer-lived products.

## Background

Current estimates of greenhouse gas (GHG) emissions from wood harvested in British Columbia (BC) may be too high because the default international accounting rules assume the biogenic carbon (C) is emitted at the time of harvest [1]. The national and provincial GHG Inventory reports follow this rule, and therefore include all biogenic C in harvested wood as an immediate emission of CO_2_[2,3]. In addition, forest C emitted as methane in landfills is reported in the waste category. Detailed accounting the C balance in harvested wood products (HWP) is important for evaluating climate change mitigation strategies. Forest ecosystems and products can contribute to mitigation efforts because the growing forest is a sink for CO_2_ and some products can store that C for a long time [4]. Furthermore, wood products have smaller GHG-footprints than other building materials [5,6], while global demand for housing continues to grow. The Government of BC has made a commitment to reduce the provincial GHG emissions; however, the forestry sector’s ability to participate in C-offset trading is limited by the methods used to account for GHG emissions.

Researchers have estimated the flow of C in HWP since the 1990s [7]. In 2006 the Intergovernmental Panel on Climate Change (IPCC) published accounting guidelines and example parameters [8]. Over the past 15 years, there has been a number of HWP life cycle analyses; particularly around building materials [9-11]. There have also been advancements made in the estimation of North America consumption and disposal of wood products [12,13] and emissions from landfills [14-16] that could be brought together in a new model. One opportunity to improve previous HWP modelling efforts includes adding to the empirical datasets on the life spans of buildings in North America. Furthermore, the parameters required to simulate wood product and paper manufacturing in BC are not publically available. Given the scientific advances in HWP accounting, the fact that about 40% of the harvested C is converted into long-lived products, and the increase in the rate of logging over time [17], I hypothesized that the annual BC GHG emissions are actually much lower than the reported emissions. The forest C offset protocol for BC recognizes 100-year long storage [18]. However, an annual or period-based estimation of storage and emissions from harvested wood products over their life cycle would improve the quality of estimates of emissions towards what the atmosphere actually receives.

This research was undertaken to improve estimates of C-storage and GHG emissions from wood harvested in BC to allow better use of forests and wood products in climate change mitigation efforts. This paper describes a new model to estimate annual net additions to C held in HWP in use and in landfills for wood harvested from forests in British Columbia from 1965 to 2065 and provides associated estimates of annual emissions to the atmosphere. The results section describes the new model including all parameters on BC manufacturing yield and waste handling over time, building life-spans, and North America market and disposal conditions. The results, discussion, and conclusion sections demonstrate the model behaviour, compares the estimates of C stocks and fluxes with similar data and models, and recommends next steps. The methods section at the end details the datasets used for input and parameterization of the model, and describes the verification, uncertainty and sensitivity methods.

## Results and discussion

### General model characteristics

**Figure 1:**
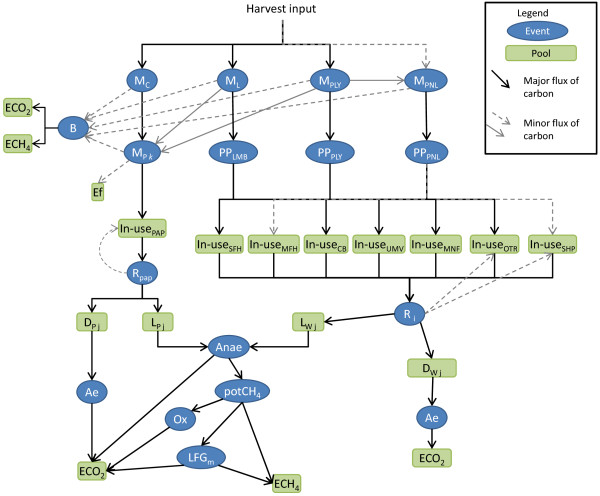
**Simplified illustration of the BC-HWPv1.** Note some lifecycle processes, stages and pools were removed from the diagram for clarity. Not all fluxes occur in all years. See Tables 1 and 2 for descriptions of abbreviations.

The British Columbia Harvested Wood Products Model version 1 (BC-HWPv1) starts with whitewood harvest as input and then simulates primary milling, construction and secondary manufacturing, retirement from material in-use, disposal and decay (Figure1). It generally follows the Production accounting method defined by the IPCC[8], however, in addition to CO_2_ emissions from HWP, it also tracks CH_4_. Only the stocks and fluxes of the C in the harvested wood are estimated. For each year from 1965–2065 the model tracks the amount of C added to or removed from various pools or reservoirs. (See the Methods section below for a description of the input dataset). There are 17 C pools in the model where C is stored for at least one year (Table1). The BC-HWPv1 uses over 17 life cycle processes or stages to transfer C between pools within a time step (Table2). The annual GHG emissions are estimated from the C stock changes in the pools representing emissions (ECO_2_ and ECH_4_) (see Methods for details). The decision to start the simulation with logs, (rather than products as is done in some other studies [19]), allows the statistics on commodity manufacturing to be used for testing the simulation parameters. If the statistics were used, assumptions would still need to be made with respect to waste disposal practices during manufacturing. One advantage with starting with the logs is the ability to simulate changes in manufacturing technology, fibre flow, and regulations in the past and into the future. I used exponential decay to describe the retirement of C from the in-use pools and decay from waste pools over time. The generic form the retention rate *(ret)* for each pool (*p*) where C is the mass of C, t is the time step, and HL is the half-life:

(1) $${{\text{C}}_{\text{t+}1}/{\text{C}}_{\text{t}}\text{=e}}^{-\text{In}\left(2\right)/\text{HL}}{\text{=ret}}_{\text{p}}$$

**Table 1:** Descriptions of the pools in the BC-HWPv1

**Pool name**	**Description**
Paper (In-use_PAP_)	Various paper and paper board products.
Single family homes (In-use_SFH_)	Long-lived building elements made from lumber, plywood and panels in single-family homes.
Multi-family homes (In-use _MFH_)	Long-lived building elements made from lumber, plywood and panels in multi-family homes.
Commercial buildings (In-use_CB_)	Long-lived building elements made from lumber, plywood and panels in commercial, industrial and public buildings.
Residential upkeep and moveable homes (In-use _UMV_)	Medium-lived building elements made from lumber, plywood and panels, (e.g. decks, fences, repairs and renovations to interior building elements). Moveable homes including mobile homes and floating homes.
Furniture & other manufacturing products (In-use_MNF_)	Products made through secondary manufacturing from lumber, plywood and panels, (e.g. furniture, guitars, decorations)
Shipping (In-use_SHP_)	Wooden containers, pallets, dunnage, blocking and bracing.
Other (In-use_OTR_)	Remainder of wood consumption
Effluent (Ef)	Pulp discarded to decay aerobically.
Dump wood (D_W_)	Wood discarded to dumps or otherwise decaying aerobically.
Dump paper (D_P_)	Paper discarded to dumps or otherwise decaying aerobically.
Degradable landfill wood (L _WD_)	Portion of wood discarded to managed, sanitary landfills that will decay anaerobically.
Non-degradable landfill wood (L _WN_)	Portion of wood discarded to managed, sanitary landfills that will not decay.
Degradable landfill paper (L_PD_)	Portion of paper discarded to managed, sanitary landfills that will decay anaerobically.
Non-degradable landfill paper (L _PN_)	Portion of paper discarded to managed, sanitary landfills that will not decay.
Emissions as C dioxide (ECO_2_)	The amount of C estimated to be released as carbon dioxide.
Emissions as methane (ECH_4_)	The amount of C estimated to be released as methane.

**Table 2:** Descriptions of the lifecycle processes and stages in BC-HWPv1

**Event name**	**Description**
Lumber mills (M_LMB_)	Transfer of C from whole logs primarily into solid wood products over 6 mm in thickness.
Chip mills (M_C_)	Transfer of whole logs primarily into chips for pulp and paper.
Plywood and veneer mills (M_PLY_)	Transfer of whole logs primarily into laminated veneer lumber, veneer and plywood.
Panel mills (M_PNL_)	Transfer of C that may come from whole logs for oriented strand board or as wood waste from other mills for use in particleboard or fibre board.
Lumber (PP_LMB_)	C in dimensional lumber.
Chips (PP_CHP_)	C in chips, predominantly softwood.
Plywood (PP_PLY_)	C in plywood with plys of 6 mm or less in thickness and veneer of 6 mm or less in thickness.
Panels (PP_PNL_)	C in oriented strand board, fiberboard, particleboard and panels.
Mechanical mills (M_P M_)	C in chips input to mechanical and semi-chemical pulping simulation
Chemical mills (M_P C_)	C in chips input to chemical pulping technologies simulation
Combustion fuel (B)	C as chips, solid wood, sawdust, black-liquor, waste wood or paper intended to be burned.
Retirement (R)	Loss of C from in-use pools into waste or recycled back into in-use pools.
Aerobic decay (Ae)	Decomposition in an oxygen-rich environment.
Anaerobic decay (Anae)	Decomposition without oxygen.
Potential CH_4_ released (pot CH_4_)	The amount of methane produced by the simulation of anaerobic decay.
Oxidation (O)	A molecular reaction where hydrogen is lost and oxygen is added.
LFG_M_	Landfill gas collection and flaring

The BC-HWPv1 works within the pool and flow capabilities of the C Budget Model Framework for Harvested Wood Products (CBMF-HWP) software. This software is a C mass-balance dynamics modelling framework currently under development by the C Accounting Team of the Canadian Forest Service (Werner Kurz, Mark Hafer and Michael Magnan personal communication). This software provides a set of basic building blocks with defined behaviour from which users may describe and parameterize mass flow and transformation networks of arbitrary complexity. A keyword-based modelling language is used to define all characteristics of the system to be studied, including spatial and temporal resolution, C storage pools, flow pathways, and controls on the flow of mass through the system; the CBMF-HWP software then reads, validates, simulates, and reports on the C dynamics of the system. The flexible nature of the framework allows the same software program to accommodate a wide variety of different analytical goals, scales and data sources.

North America was treated as one spatial area in the modeling framework because 90% of BC harvested C remains in Canada and the USA. The best available literature for wood product in-use and disposal are also from the United States. Furthermore, the two countries are similar in wood product markets and culture. The BC-HWPv1 assumed exports of lumber and other products to regions outside of Canada and the USA follow the same life cycle, because of lack information and small quantity of exports outside of North America.

The BC-HWPv1 is similar to a bookkeeping model relying only on addition, subtraction, and multiplication. The complexity in BC-HWPv1 is not in the math, but in the detail of life cycle processes, stages, and the changing parameter values over time. For example, Equation 2 describes the annual change in the amount of wood C in single family homes (In-use_SFH_) as a function of the annual harvest input, the flow of C through the mills, into primary products and into the single family home pool, minus the amount of C retired from use. A second example describes how the model simulates the concept of paper recycling (Equation 3). Each parameter set is explained below.

ΔIn-use_SFH_ = (H x pM_LMB_ x P_LMB_ x S_LMB_) + (H x pM_PLY_ x P_PLY_ x S_PLY_) + ((H x pM_PNL_) + (H x pM_PLY_ x pM_PLY-PNL_)) x (P_PNL_ x S_PNL_) – (In-use_SFH, t-1_ x (1- ret_SFH_)) (2)

H = annual harvest

pM_h_ = proportion of harvest sent to each different wood product mill, (subscripts LMB = lumber, PLY = plywood, PNL = panel), see below.

pM_PLY-PNL_ = proportion of C from the plywood mills that gets transferred to panel mills, see below.

P_g_ = proportion of C from each wood product mills that is transferred to each primary product, (subscripts LMB = lumber, PLY = plywood, PNL = panel), see below.

S_i_ = proportion of each primary product that is made into single family homes, (subscripts LMB = lumber, PLY = plywood, PNL = panel), see below.

ret_SFH_ = annual retention rate for all wood products in single family homes.

ΔIn-use_PAP_ = [(H x pM_h_ x P_CHP_ x pM_P k_ x p_k_) + (ΔIn-use_PAP, t-1_ x (1 - ret_PAP_) x y_PAP_)] – [ΔIn-use_PAP, t-1_ x (1-ret_PAP_)] (3)

ΔIn-use_PAP_ = annual change in C stocks in the In-use paper pool.

P_CHP_ = proportion of C from each wood product mills that is transferred to chips, see below.

pM_P k_ = proportion of chips sent to mechanical or chemical mills, see below.

p_k_ = proportion of C at either the mechanical or chemical mills that is made into paper, see below.

ret_PAP_ = annual retention rate for paper.

y_PAP_ = proportion of retired paper that is recycled back to paper.

### BC-HWPv1 parameters

The BC-HWPv1 divided the input C among four types of life cycle processes: lumber, chip, plywood, and panel mills (see Table2 for descriptions). In reality, the chip mills are part of the pulp mill facilities. For modelling purposes, I kept the chipping and pulping processes as separate life cycle processes because chips are sent to become pulp through various pathways. The proportion of harvest assigned to each type of mill varied over time (Table3). The model simulated manufacturing in each of the four mills. Each primary wood product – lumber, chips, plywood, or panels, had a time dependent yield (Table4). A proportion of the C also entered the combustion fuel life cycle stage, the dump wood pools or landfill wood pools. BC-HWPv1 treated biomass used for bioenergy as combustion in the year of harvest. (See the Methods section below for a description of the uncertainty and sensitivity analyses).

**Table 3:** Percent distribution of harvested C among mill life cycle processes in BC-HWPv1

**Decade**	**Lumber mills (M_LMB_)**	**Chip mills (M_C_)**	**Plywood mills (M_PLY_)**	**Panel mills (M_PNL_)**	**Sources for the time period**
1990–2065	84	5	8	3	[20]
	(82–91)	(3–7)	(6–8)	(0–4)	
1980–1989	79	13	8		Linear interpolation
	(73–84.5)	(7.8–16.5)	(7.5–10.5)		
1970–1979	72	19	9		[21,22]
	(64–78)	(12.5–26)	(9–13)		
1965–1969	76	15	9		[23]
	(75–76.8)	(14.6–15.5)	(8.6–9.2)		

Brackets include the minimum and maximum in the underlying source data. Most of the variability was between years.

**Table 4:** Percent distribution of C into primary wood products life cycle stages and disposal pools for British Columbia

**Time period**	**To**	**From**			
		**Lumber mills (M_LMB_)**	**Chip mills (M_C_)**	**Plywood mills (M_PLY_)**	**Panel mills (M_PNL_)**
1995-2065	Lumber (PP_LMB_)	47			
		(45–56)			
	Plywood (PP_PLY_)			51	
				(51–56)	
	Panels (PP_PNL_)				84
					(73–95)
	Chips (PP_CHP_)	35	96.3	24	
		(26–38)		(22–27)	
	Panels mills (M_PLY-PNL_)	0		16	
		(0–11)		(10–17)	
	Combustion fuel (B)	17.9	3.2	8.5	15.5
		(4–18)		(6–13)	(4.5–23)
	Landfill wood (L_W j_)	0.1	0.5	0.5	0.5
		(0–1)		(0–1)	(0–0.5)
Sources for the time period	[24]	[[20,25-27], B Lippke 2010 pers comm.]	[28]	[9,29,30]	[31-33]
1980–1994	Lumber (PP_LMB_)	44			
	Plywood (PP_PLY_)			50	
	Chips (PP_CHP_)	32	78	16	
				(16–41)	
	Combustion fuel (B)	23	21	32	
				(7–32)	
	Dump wood (D_W_)	1	1	2	
Sources for the time period		[25]	Linear interpolation	[21,30,34-36]	
1965–1979	Lumber (PP_LMB_)	40			
		(30–47)			
	Plywood (PP_PLY_)			50	
	Chips (PP_CHP_)	29	60	16	
		(20–40)	(53–84)	(16–41)	
	Combustion fuel (B)	29	38	32	
		(18–36)	(14–45)	(7–32)	
	Dump wood (D_W_)	2	2	2	
Sources for the time period		[21,30,35-38]	[34]	[21,30,34-36]	

Brackets include the minimum and maximum in the underlying source data.

After BC-HWPv1 had estimated the C in chips for a given year, it distributed the C between mechanical and semi-chemical mill or chemical mill life cycle processes (Table5). The model calculated the amount of C to add to the paper in-use pool based on the yield (Table6). The model sent the remainder of the C from the mechanical and chemical mill stages to either the combustion fuel stage or the effluent pool. The model transferred half of the waste from the chemical mill stage to the effluent pool from 1965–79 (Table6). Starting in 1980, the model sent very little C to the effluent pool due to changes to the waste handling regulations [24]. 

**Table 5:** Percent of chips sent to mechanical or chemical mill life cycle processes

**Decade**	**Mechanical mills (M_P M_)**	**Chemical mills (M_P C_)**	**Sources for the time period**
2000–2065	12	88	[39]
	(6–14)	(86–94)	
1990–1999	16	84	[39]
	(13–20)	(80–87)	
1980–1989	18	82	[39]
	(17–19)	(81–83)	
1965–1979	30	70	[40]

Brackets include the minimum and maximum in the underlying source data. The variability was between years.

**Table 6:** Pulp yield and disposal of residues for BC as percentages of input

		**From life cycle process**		
**Time period**	**To pools**	**Mechanical mills (M_P M_)**	Chemical mills (M_P C_)*	**Sources for the time period**
1980–2065	Paper (In-use_PAP_)	93	45	[28,41,42]
		(92–95)	(30 – 45)	
	Combustion fuel (B)	6.9	53.9	
	Effluent (Ef)	0.1	1.1	
1965–1979	Paper (In-use_PAP_)	95	38	[43,44]
		(92–95)	(30 – 40)	
	Combustion fuel (B)		31	
	Effluent (Ef)	5	31	

*Note, 10% reduction of C due to removal of lignin [45].

The BC-HWPv1 simulated the construction and manufacturing from lumber, plywood and panels into in-use products. BC-HWPv1 added C from each type of primary wood product life cycle stages to the in-use pools, dump wood pools, landfill wood pools or recycled life cycle process using predefined proportions (Table7). Throughout the model, BC-HWPv1 handled recycling as a transfer of C to the other pool unless coming from the paper or shipping pools, which are recycled back to themselves because of their short half-lives compared to the other pool.

**Table 7:** **The percent distribution of C from primary wood products stages to in-use pools**[12,46,47]

		**From**		
**Time period**	**To pools**	**Lumber**	**Plywood**	**Panels**
1990–2065	Single family homes (In-use_SFH_)	25	41	15
		(21–29)	(34–44)	(11–19.5)
	Multi-family homes (In-use_MFH_)	1.5	3	2
		(1–2.5)	(2–4)	(1–2.3)
	Commercial buildings (In-use _CB_)	7	9	6
		(5.5–8.5)	(7–11)	(5–7)
	Residential upkeep and moveable homes (In-use_UMV_)	25	25.5	16
		(21–28)	(21–33)	(11–24)
	Furniture & other manufacturing products (In-use_MNF_)	10	7.5	36
		(6.5–12)	(5.5–10)	(23–47)
	Shipping (In-use_SHP_)	10	2	1
		(9–11)	(1.5–2)	(0.4–1)
	Other (In-use_OTR_)	12	7	19
		(8–16)	(5.5–10.5)	(5–28)
	Landfill wood (L _W j_)	7.5	4	4
	Recycle (In-use_OTR_)	2	1	1
1965–1989	Single family homes (In-use_SFH_)	26	34	
		(20–36)	(26–47)	
	Multi-family homes (In-use_MFH_)	5.5	8	
		(3–11.5)	(4–14)	
	Commercial buildings (In-use_CB_)	10	13	
		(7.5–12.5)	(9–18)	
	Residential upkeep and moveable homes (In-use_UMV_)	15	20	
		(9–27)	(14–28.5)	
	Furniture & other manufacturing products (In-use _MNF_)	11	7	
		(8.5–13)	(5–9)	
	Shipping (In-use_SHP_)	12	2	
		(9–16)	(1.5–2.5)	
	Other (In-use_OTR_)	10.5	11	
		(2–21)	(5.5–19)	
	Landfill wood (L_W j_)	10	5	

Brackets include the minimum and maximum in the annual data provided by McKeever [47].

Based on the available information, the half-lives for the C in-use pools ranged from 2 to 90 years in BC-HWPv1 (Table8). The minimum and maximum half-lives used in the uncertainty analysis represent the range of possible values found in the literature and datasets, or, in the case of the maximum for buildings, an arbitrary value, since the maximum half-life calculated was many multiples of the number of years covered by the data.

**Table 8:** Half-lives assumed for in-use pools in BC-HWPv1

**Pools for harvested wood products in-use**	**Assumed Half-life in BC-HWP v1**	**Source (Data presented in Methods section)**
Single-family homes	90	[[13,48,49] and this study]
	(78–350)	
Multi-family homes and commercial buildings	75	[[13,48,50,51] and this study]
	(48–350)	
Residential upkeep and moveable homes	30	[[10,13,52,53] and this study]
	(5–50)	
Furniture & other manufactured products	38	[[10,13,52,53] and this study]
	(19–38)	
Shipping	2	E. Allen, B. Eggertson, B. Scholnick Personal communication
	(1–2)	
Other (Remainder of wood consumption)	38	[13]
	(19–38)	
Paper	2.5	[13]

Brackets include the minimum and maximum used in the uncertainty analysis.

Shipping requires 8.6% of the C from primary products stages (Table7). The pallet and container industry seems to collect data on the lifespan of wooden pallets and containers by assessing the number of loads rather than years, so I relied on expert opinions to determine the half-life.

As HWP are retired from use, the C may be recycled back to an in-use pool, sent to the combustion fuel life-cycle stage, to a dump pool, or to the landfill pools; parameters follow from [13]. (Refer to Figure1) I made an exception for the amount of paper recycled back into paper, of the 50% of disposed paper that was recycled, 8% was estimated to be unrecoverable [28]. I also assumed future disposal parameters would remain the same as in 2005. The BC-HWPv1 recycled wood from the in-use pools to the other products pool except for shipping which was recycled back to itself.

The BC-HWPv1 assumed C in the dump pools and the effluent pool will completely decay through aerobic processes and sent the C to the emissions as C dioxide pool. The retention rate depended on the pool [13]. The BC-HWPv1 split HWP in landfills into degradable and non-degradable pools [13,14]. The proportion degradable depended on whether the material was wood (23%) or paper (56%). The model simulated the anaerobic decay of the degradable pools over time with 50% of the C sent to the emissions as C dioxide pool and 50% assumed to be methane [14].

The amount of methane produced by the simulation of anaerobic decay is the potential CH_4_ released (*potCH*_*4*_). However, landfill gas management efforts ensure that not all of that methane is released. The BC-HWPv1 estimated methane emissions by multiplying the *potCH*_*4*_ by the proportion of methane produced at landfills without a gas collection system, the efficiency of the collection system, and the oxidation rate through the landfill cap (Equation 6). Based on EPA data [16,54-56] on the percent of the methane produced at landfills with gas collection systems, I derived a linear regression of percent over time (r^2^ = 0.88 and *P* = 0.064). This analysis predicted the increased adoption of landfill capture systems from 0 in 1988 to 98% in 2015 (Table9). The landfill gas collection systems were modelled with 75% capture efficiency from 1990 until 2007 [55] and 87% efficiency from 2008 to the end of the simulation [16]. Together, these two parameters describe the effect of landfill gas management over time as increasing from 0 to 85% net reduction in CH_4_. Once captured, the BC-HWPv1 simulated the methane as burned and the C added to the emissions as carbon dioxide pool. Of the C remaining methane, the model added a proportion to the emissions as carbon dioxide pool to represent oxidation through the landfill cap and the rest was added to the emissions as methane pool. 

**Table 9:** The changes in landfill gas management as modelled in BC-HWPv1

**Time period**	**Methane produced at landfills with capture technology (%)**	**Sources for the technology at landfills**	**Capture efficiency (%) [**[16]**] [**[55]**]**	**Net reduction in CH**_4_ d**ue to landfill gas management (%)**
2015 -2115	98	Regression	87	85
2011-2014	82	Regression	87	71
2008-2010	65	[16]	87	57
2003-2007	59	[56]	75	44
2000-2002	49	[55]	75	37
1995-1999	17	[54]	75	13
1990-1994	5	Regression	75	4
1965-1989	0		0	0

Chanton and others [15] reviewed the literature of studies estimating the methane oxidation in landfill cover soils. From their review, nine studies met the following two criteria: using landfill cover material and, the studies occurred over the entire year, because these are more likely to reflect operational conditions. Based on the median from the subset of studies, the BC-HWPv1 used 22% for the fraction of methane oxidized through the landfill cover. The uncertainty analysis used the range of 10 to 84%. (See the Methods section below for more information on the uncertainty and sensitivity analyses).

Combustion of wood and paper C relied on the same methods as the Canadian inventory of greenhouse gas for industrial wood boilers [3]. The BC-HWPv1 moved the C from the combustion fuel stage (B) to the emissions as C dioxide and emissions as methane pools as described in Equations 4 and 5. Although in reality these are fluxes, the *ECO*_*2*_ and *ECH*_*4*_ function as pools in the BC-HWPv1.

(2) $$\text{annual increase to}\;ECO_{2}=0.9999985\times B$$

(3) $$\text{annual increase to}\;ECO_{4}=0.0000015\times B$$

The annual change in the *ECH*_*4*_ was the accumulation of C from burning of waste from mill and retirement life cycle processes, plus the anaerobic decomposition in landfills as simulated by BC-HWPv1 (Equation 6).

(4) $$\Delta {\text{ECH}}_{4}\text{ = }\left(0\text{.0000015xB}\right)\text{+ }\left[{\text{L}}_{\text{PD t}-1}\text{x }\left({1-\text{ ret}}_{\text{LPD}}\right)\text{ x }0\text{.5 x }\left(\left({1-\text{LFG}}_{\text{F}}\right)\text{ - }\left({1-\text{LFG}}_{\text{F}}\right)\text{ x O}\right)\right]\text{+ }\left[{\text{L}}_{\text{PD t}-1}\text{x }\left({1-\text{ ret}}_{\text{LPD}}\right)\text{ x }0{\text{.5 x LFG}}_{\text{F}}\text{x }\left(\left({1-\text{LFG}}_{\text{e}}\right)\text{ - }\left({1-\text{LFG}}_{\text{e}}\right)\text{ x O}\right)\right]\text{+ }\left[{\text{L}}_{\text{WD t}-1}\text{x }\left({1-\text{ ret}}_{\text{LWD}}\right)\text{ x }0\text{.5 x }\left(\left({1-\text{LFG}}_{\text{F}}\right)\text{ - }\left({1-\text{LFG}}_{\text{F}}\right)\text{ x O}\right)\right]\text{+ }\left[{\text{L}}_{\text{WD t}-1}\text{x }\left({1-\text{ ret}}_{\text{LWD}}\right)\text{ x }0{\text{.5 x LFG}}_{\text{F}}\text{x }\left(\left({1-\text{LFG}}_{\text{e}}\right)\text{ - }\left({1-\text{LFG}}_{\text{e}}\right)\text{ x O}\right)\right]$$

Where:

L_PD t-1_ = the amount of C in the degradable landfill paper pool

ret_LPD_ = annual retention rate for landfill paper

LFG_F_ = proportion of methane produced at landfills with gas collection systems

O = the proportion of methane oxidized to CO_2_ by the landfill cover material

LFG_e_ = landfill gas collection system efficiency

L_WD t-1_ = the amount of C in the degradable landfill wood pool

ret_LWD_ = annual retention rate for landfill wood

See the Methods section below for details on GHG emission calculations.

### BC-HWPv1 estimates of carbon stocks

This section demonstrates the model behaviour and outputs starting with the C in primary products (lumber, plywood and veneer, panels, and paper) estimated annually, and then the C stored in use or in waste disposal sites (refer to Figure1). Where available, I compared the model results with commodity statistics or other published models. See the Methods section below for more information on the input data, verification, uncertainty and sensitivity analyses.

**Figure 2:**
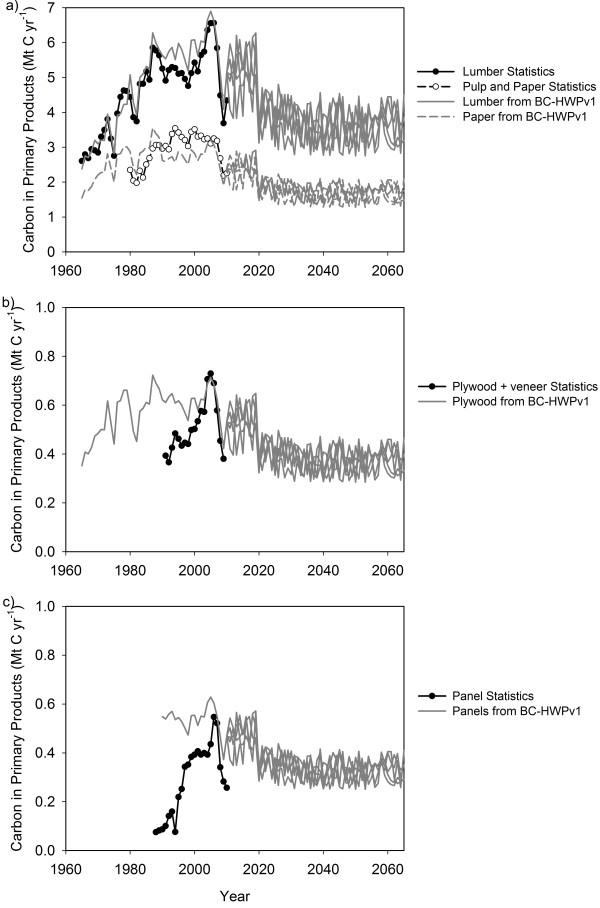
**Comparison of the estimates of C in primary wood product stages by BC-HWPv1 and commodity statistics.a**) Lumber and paper, **b**) plywood and **c**) panels. Note the y-axes vary. Future harvests varied between 61 and 100% of the estimated sustainable timber flow to reflect the uncertainty in forecasting.

The BC-HWPv1 estimated that the amount of C in manufactured products generally increased from 1965 to 2005 due to increased harvesting (Figure2). The steep decline in harvest in 2007–09 and lower harvest rates in the future translated directly into lower amounts of C in products in the simulation results. The amount of C in the lumber and paper life cycle stages from the BC-HWPv1 simulation generally agreed with the available commodity statistics (Figure2a). There was greater inter-annual variability in the statistics because the manufacturers will change their output quickly, whereas the model parameters are set to change on a decadal or longer basis. Some of the differences are also due to the export of logs and chips which are therefore manufactured into products in a different jurisdiction. The difference between lumber in the simulation and the statistics ranged from −0.48 to +0.942 Mt C with a median difference of 0.275 Mt C over 46 years. For paper, the difference ranged from −0.975 to +0.759 Mt C with a median of −0.287 Mt C over 31 years.

The amount of C in the plywood life cycle stage from the BC-HWPv1 simulation was quite similar to commodity statistics in the 2000s (Figure2b). The BC forest industry has been producing high quality veneers, furniture-grade plywood, and construction sheathing since the early 1900s [40]. The mill survey statistics from 1990–2009 include veneer and all plywood products; however they are based on a voluntary survey and may be incomplete [20]. The difference between the simulated plywood C and the commodity statistics for plywood plus veneer ranged from −0.018 to +0.268 Mt C with a median of +0.096 Mt C over 19 years. Similar to plywood, the commodity statistics for panels relied on a voluntary survey and may be incomplete [20]. The difference between the simulated panel C and the statistics for panels ranged from −0.82 to +0.462 Mt C with a median of +0.161 Mt C over 23 years (Figure2c). Some of the differences are likely also due to the export of logs, chips, and sawdust (see Methods).

The manufacturing simulated by the BC-HWPv1 produced substantially higher emissions than the CORRIM 2005 manufacturing (cradle-to-gate) wood utilization model [57], despite relying on many of the same publications. The key difference appears to be that the CORRIM wood utilization sent about 50% of the harvested white-wood to lumber, about 50% to short-lived products with a loss rate of 10% per year and only bark was burned. However, the BC-HWPv1 estimated 35% of the white-wood burned; bark was not tracked. Bark is approximately an additional 11% of the biomass of the stemwood [58]. The difference in the initial distribution of white-wood into short-lived products rather than combustion is largely responsible for the lower emissions in the CORRIM study. Both the CORRIM and the BC-HWPv1 estimates of C loss during manufacturing are lower than other production-based estimates [7,13,59].

**Figure 3:**
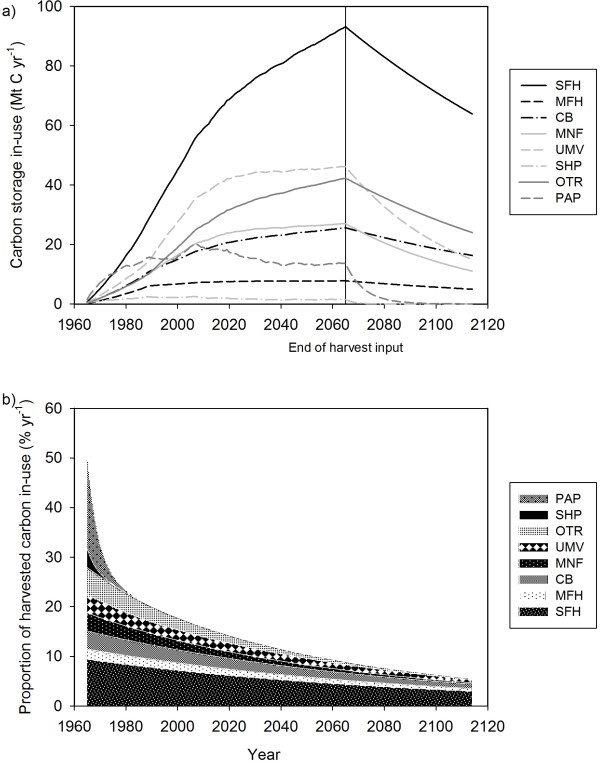
**Estimates of C stored in in-use pools over one of the 150 year simulations: single family homes (SFH), multi-family homes (MFH), commercial buildings (CB), furniture and manufactured products (MNF), residential upkeep and moveable homes (UMV), shipping (SHP), other wood products (OTR), and paper (PAP).a**) Annual C stocks in all in-use pools. Note that harvest and therefore input to these pools occurred from 1965 to 2065 only to demonstrate the different rates of loss after 2065. **b**) The decline of C stored in-use that originated from the 1965 harvest only.

The C stored in-use pools increased at the beginning of the simulation; in part because of the increasing rate of harvest for the first 40 years of the simulation (Figure3a). In addition, the pools need to be “filled up” until there were stocks of a sufficient age to be lost through retirement. At the end of simulated harvest in 2065, the single-family housing pool had the greatest amount of C stored in-use due to both the large proportion of products entering that pool and it having the longest half-life at 90 years. Residential upkeep and moveable homes had the second highest stocks in 2065, despite one of the lowest half-lives of 30 years. After 2065, as input to the in-use pools stopped, the different steepness in decline reflects the different half-life parameters for most pools.

The cumulative storage of C in-use from the 1965 harvest showed a steep decline in the first 15 years (Figure3b). The BC-HWPv1 estimated that after 150 years, 5.5% of the harvest was still in-use, primarily in single-family homes.

**Figure 4:**
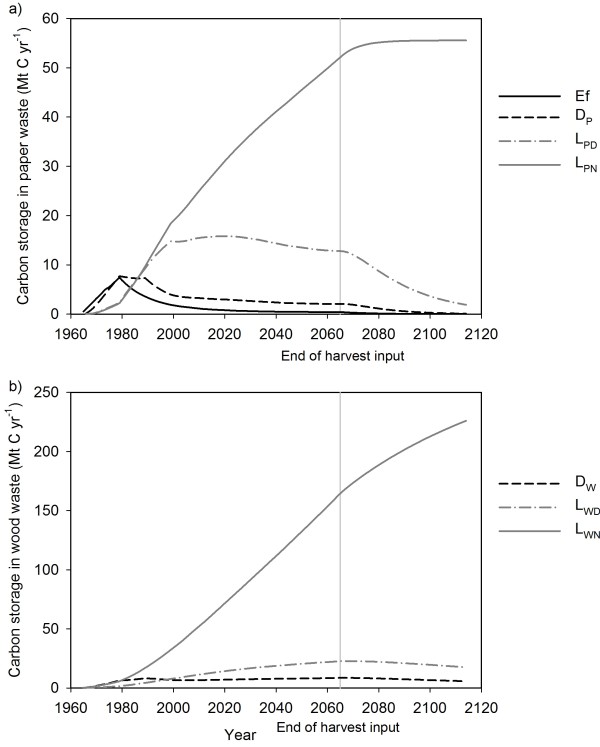
**Estimates of C stored in waste pools over one of the 150 year simulations.a**) Annual C stocks of paper waste as effluent (Ef), dump paper (DP), landfill degradable paper (LPD) and landfill non-degradable paper (LPN). **b**) Annual C stocks of wood waste as dump wood (DW), landfill degradable wood (LWD) and landfill non-degradable wood (LWN). Note that harvest occurred from 1965 to 2065 only, however input to these pools from the in-use pools continued until 2110.

The dynamics of C storage in disposal sites illustrate the changing waste management practices over time, increasing harvest rates and an initialization artifact (Figure4). The amount of C in the effluent, dump paper and the dump wood pools were highest in the first few decades of the simulation. C in dumps and effluent were assumed to decay aerobically. As waste parameters changed over time, the BC-HWPv1 sent more C to either combustion fuel or landfill pools. The BC-HWPv1 simulated anaerobic decay of degradable C in landfills. The largest C stocks were in the non-degradable landfill pools as they only accumulate C during the simulation.

The cumulative amount of harvested C stored provides one metric for comparing different models of HWP in North America, although time periods can vary. The BC-HWPv1 estimate for the percent of cumulative harvest stored from 1965–2010 was 48%. This rate is similar to the 43% storage of harvest from 1920–86 for a Canadian national study [60], but significantly higher than the 23% storage of harvest from 1920–92 in Oregon and Washington [7]. The higher rates were due to the incorporation of non-degradable dynamics in landfills. The decision to include landfill dynamics and waste management in general is dependent on the question the model addresses.

The annual net C balance (net accumulation) provides a second metric to compare models of HWP in North America. In 2005–2010, the BC-HWPv1 estimated the stock change of stored C to be 46% of harvest per year (25% in use and 21% in landfills and dumps). This estimate is similar to the net accumulation from the WOODCARB 1 that I estimated at 42% net accumulation per year based on published model results. Specifically, I used the stock change values from Skog and Nicholson [61] and Woodbury and others [62] and the assumption of 139 Mt C harvested in 1991 [63]. Both BC-HWPv1 and WOODCARB1 simulated the accumulation of more C than the WOODCARB 2 [13]. I estimated 23-27% net accumulation for WOODCARB 2 using the assumption of 129 – 145 Mt C harvested per year [63]. In contrast, the BC-HWPv1 accumulation rates were lower than the approximately 60% of harvest per year as modelled by the FORCARB-ON for the province of Ontario [64].

### GHG emissions from harvested wood C

This section provides estimates of GHG emissions based on the tracking of C stock changes. The discussion includes both the backward-looking form of accounting as is used for GHG inventories and a future-focused accounting for C-offsets. It also includes comparisons with other estimates and a novel approach to a simplified accounting method. The last part of this section provides the results of the uncertainty analysis described in the Methods section.

**Figure 5:**
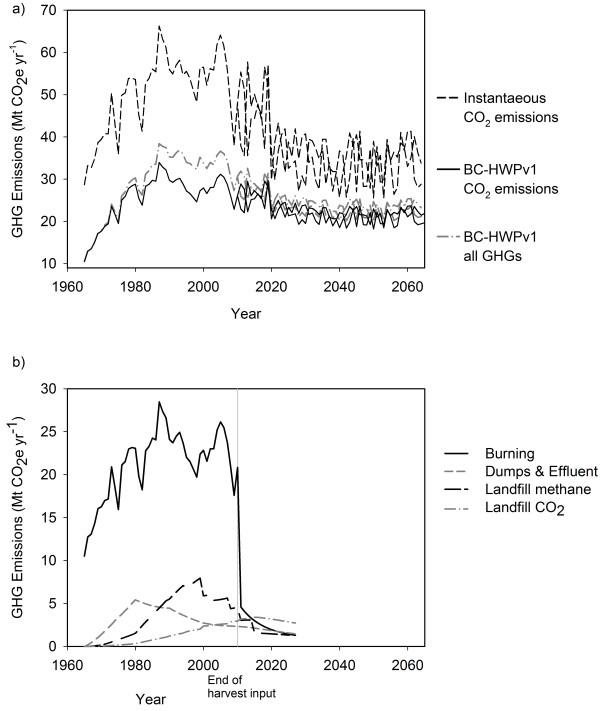
**a) Annual greenhouse gas emissions estimates from harvested C converted to CO**_**2**_**(immediate emissions), the C flux from emissions as CO**_**2**_**reported by the BC-HWPv1, or all greenhouse gas emissions (including CH**_**4**_**and N**_**2**_**O) as estimated from the C stock changes.** Only two of the future harvest forecasts are included in the graph to provide clarity. Future harvests varied between 61 and 100% of the estimated sustainable timber flow to reflect the uncertainty in forecasting. **b**) Annual greenhouse gas emissions broken down by source in BC-HWPv1 from 1965–2030. Note that harvest input stopped in 2010 in this simulation to separate burning at mills from burning at retirement. The sum of emissions in (**b**) is identical to the “BC-HWPv1 all GHGs” line in (**a**) from 1965 – 2010.

The net stock change in C can be used to estimate the GHG emissions to the atmosphere. The annual CO_2_–only emissions estimated by the BC-HWPv1 increased from 10.5 Mt CO_2_e in 1965 to a maximum of 38 Mt CO_2_e in 1987 (Figure5a). The simulation underestimated emissions during the early years because the dumps and landfill pools were initially empty. The peak 1987 emissions of all GHGs included CO_2_ from decay and burning (34 Mt CO_2_) plus methane and nitrous oxide from burning and landfill gas (4 Mt CO_2_e) (Figure5b). If I assumed instantaneous emissions of harvested C, then the estimate of 1987 emissions would be 66 Mt CO_2_e. Both Canada and BC use the IPCC default rule of instantaneous emissions for greenhouse inventories [1].

The emission sources were greatest from the combustion during the primary milling simulation of the BC-HWPv1, peaking at 28 Mt CO_2_e in 1987 (Figure5b). In this graph the milling emissions end in 2010 for illustration purposes only. Combustion emissions after 2010 represent the disposal from in-use pools. Because the largest source of emissions was from burning of mill residues, then this is the most likely area for mitigation activity. Some of the combustion is used for bioenergy instead of natural gas, however there is a proportion of the wood that is simply burned as a waste management tool [personal observation]. One potential opportunity is for more efficient energy use at the mills. A second opportunity is divert the waste wood C not being used for energy C from boilers to other wood products with longer life-spans. Given that the material is likely in the form of chips, trimmings and other small pieces potential suitable uses include fibreboard or particleboard, which, when integrated into dwellings or furniture, will have a life expectancy of 30–90 years (Table8). The step-wise nature of the landfill methane emissions estimates clearly shows the beneficial impact of landfill gas management on overall GHG estimates. These landfill gas parameters in BC-HWPv1 rely on the figures for the USA. The Canadian estimates of landfill gas recovery and utilization are lower, currently at about 28% of methane production [3]. Separate modelling of landfill dynamics and management in each country would improve the estimates of GHG emissions.

The results show that instantaneous emissions method of accounting for HWP overestimates GHG emissions. For example, the HWP emissions in 2010 could be estimated at 26 Mt CO_2_ (CO_2_ only) using the BC-HWPv1, instead of 48 Mt CO_2_. This result is consistent with previous studies from Australia [65], USA [66], Canada [67] and globally [19]. The more recent guidelines for greenhouse gas inventories from the IPCC [8] does include a set of equations and a spreadsheet for annual HWP accounting. However, in the future, it may be possible for the simplest reporting tier to use an emission factor applied to the annual harvest. For example, the CO_2_-only emissions estimated from 1980–2010 by BC-HWPv1 ranged from 48% to 60% of the harvest, with a median of 52%, an average of 52% and a mode of 54%. There was no significant trend over the time period (P > 0.13). Therefore, 0.52 could be used as a simple emissions factor for BC, instead of the current 1.0. Using factors to estimate emissions is common in other sectors because of the convenience. However, it is still a simplification and lacks precision. Given the uncertainty in many of the parameters of tracking C in HWP, this may be an acceptable trade-off. As a scientific community, we could work towards developing a suite of emissions factors for different manufacturing, wood market, and disposal circumstances.

**Figure 6:**
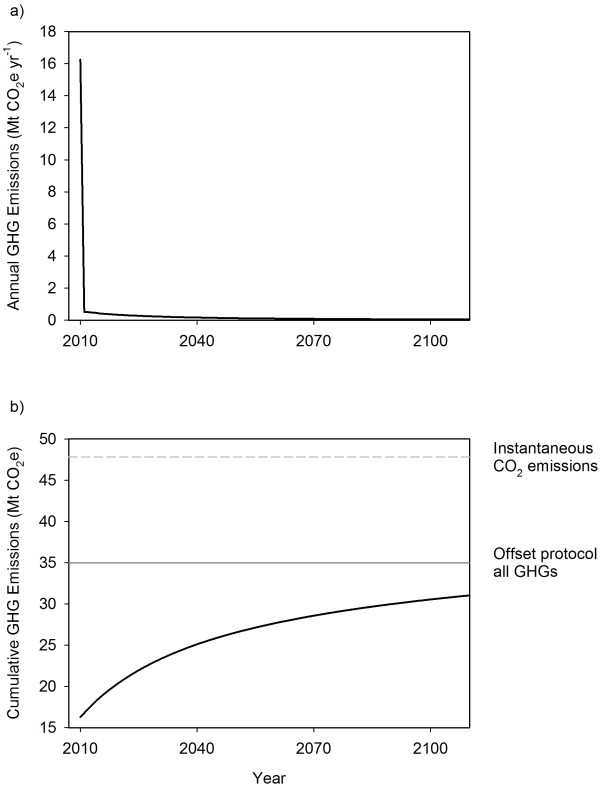
**Simulation of the greenhouse gas emissions from the harvest in 2010.a**) Annual emissions estimated using the C stock changes in BC-HWPv1 and converting to CO_2_e (including CH_4_ and N_2_O). **b**) Cumulative greenhouse gas emissions over 100 years from the 2010 harvest. The emissions are estimated from: converting the C fluxes in BC-HWPv1 (including CH_4_ and N_2_O), or converting the amount of harvested C to CO_2_ as an instantaneous emissions (IPCC default accounting), or the 100 years of cumulative emissions in the BC offset protocol for forest C (including CH_4_ and N_2_O) [18].

The GHG estimates from the BC-HWPv1 are difficult to compare with other calculations that have different system boundaries. Life cycle estimates may or may not include biogenic C dioxide release as an emission, methane and nitrous oxide from combustion or landfills, fossil fuel use, or substitution benefits [20]. The BC government forest C offset protocol has the same system boundaries but a 100-year time step instead of annual, and different pools and parameters [18]. Consider the 2010 harvest. The annual emissions are high in the first year as the BC-HWPv1 simulates milling and construction (Figure6a). The cumulative emissions over 100 years was 31 Mt CO_2_e (Figure6b). This estimate is 11% lower than what would be estimated using the offset protocol. Given the high level of uncertainty in the models, these estimates are quite similar. The cumulative emissions will never reach the total calculated from instantaneous emissions because of the storage in the non-degradable landfill pools.

**Figure 7:**
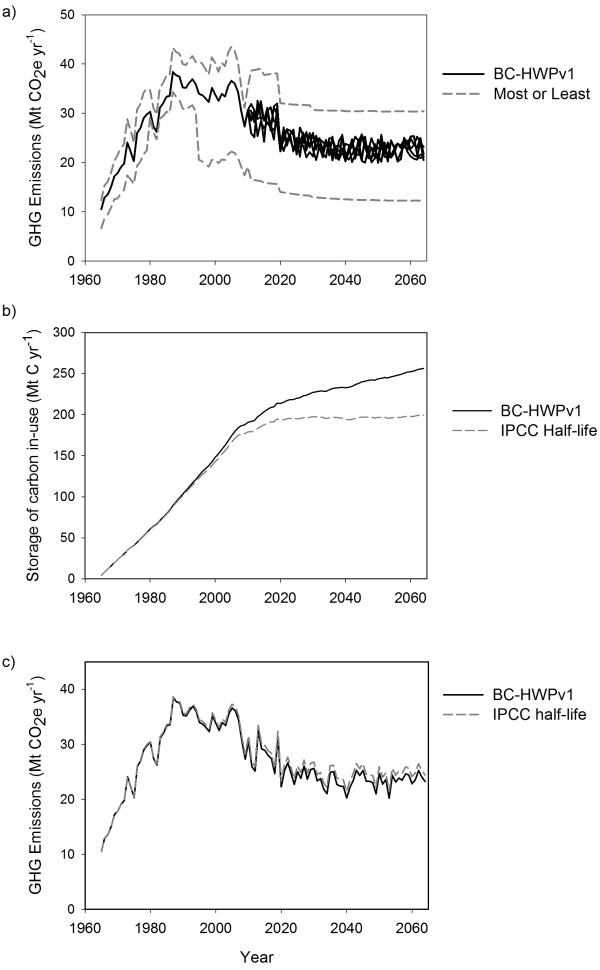
**Uncertainty analysis of parameters in the BC-HWPv1.a**) Potential uncertainty if all the parameters and future harvest scenarios were set to generate the most or the least possible greenhouse gas emissions. **b**) Annual in-use C stocks and **c**) GHG emissions when half-lives set to the IPCC suggested values, (30 years for wood products).

One source of uncertainty comes from the range of potentially valid parameter values. (See Methods section for description of uncertainty and sensitivity analyses). If we assumed all the possible parameters for the least GHG emissions, the 1965 estimate was 6.7 Mt CO_2_e. However, assuming all the highest emission parameters, it was 12 Mt CO_2_e (Figure7a). In 1987, the range was 9 Mt CO_2_e. The difference between the two estimates increased further starting in 1995 (about 20 Mt CO_2_e yr^-1^). This increase was in large part due to the assumption that 11% of the C from the lumber mills life cycle stage was transferred to the panel mills life cycle stage instead of being burned and 95% of that C was converted to panels. The sensitivity of the emissions estimate to this single transfer of C reinforces the suggestion above that reducing the amount of burning and putting that C into longer-lived products could have important climate change mitigation impacts.

When I assumed 30 years half-life for the wood product pools, the estimate of C in-use started to diverge noticeably from the BC-HWPv1 estimate after about 30 years into the simulation (Figure7b and c). These results indicate that the IPCC default half-life values are too low for estimating C storage in wood products in North America. However, the difference in GHG emission estimates was negligible because they were more sensitive to parameters for milling and waste (Figure7b and c). These results indicate that the IPCC default half-life values can continue to be relied on for log to landfill (i.e. cradle-to-grave) estimates of GHG emissions.

**Figure 8:**
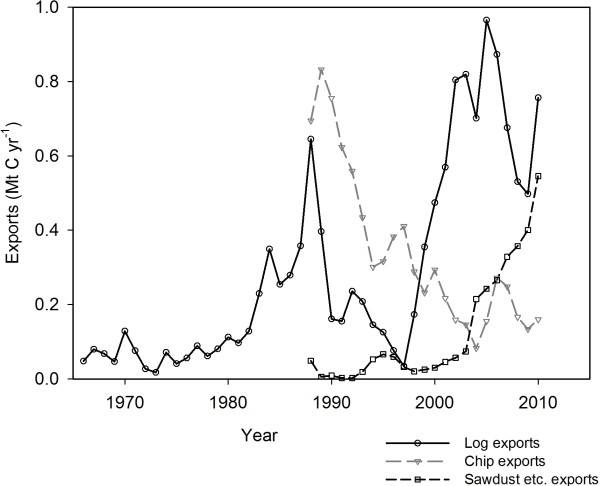
**Commodity statistics on log exports (1966–2010), chip exports and sawdust, shavings and wood waste exports (1988–2010).** Pellets and fire-bricks are likely included in the sawdust category.

Uncertainties in the BC-HWPv1 estimates of GHG emissions are also due to inter-annual variation in manufacturing, exports of logs, chips and wood products beyond the USA (Figure8), divergent parameters from the literature and data gaps on dumps and landfill management in Canada. The GHG estimates were most sensitive to uncertainties around manufacturing efficiency, mill waste handling and landfill gas capture. Therefore, these are the areas of importance for further research.

## Conclusions

This research supports early research findings that the C dynamics are highly sensitive to initial manufacturing practices and assumptions about emissions from landfills. If the Government of BC reported annual harvesting emissions using the BC-HWPv1, the estimate would be reduced by about 45% for 2010. An emissions factor of 0.52 multiplied by the annual harvest would provide a simple alternative to GHG accounting for BC. If an offset projects reported 100 year cumulative emissions using the BC-HWPv1 the emissions would be lower by about 11%, a difference likely within the uncertainty of the models. Substantial data gaps remain about the amount of wood C used for bioenergy in BC and the landfill gas management in Canada. The simulations demonstrated that the primary opportunities for climate change mitigation are in shifting mill waste from burning to longer-lived products such as fibreboard.

## Methods

### Input harvest data

**Figure 9:**
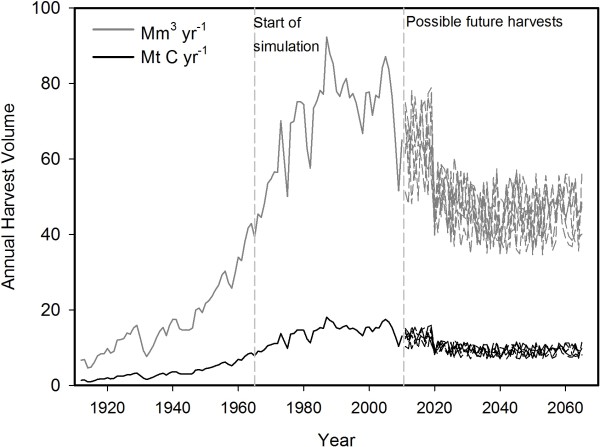
**Harvest volume in cubic metres per year (grey lines) and in tonnes of C per year (black lines) from 1912–2065.** BC-HWPv1 used the C data from 1965 to 2065 as input. There is an ensemble of six possible future harvest rates starting in 2011. Future harvests varied between 61 and 100% of the estimated sustainable timber flow to reflect the uncertainty in forecasting.

The annual amount of logged C (inside-bark) is the input data to the BC-HWPv1. The harvest data from 1912–2010 are maintained by the BC Government and available through the government website [40,68]. The roundwood volume from 1912 – 1951 was converted from foot board measure to log volume using the factors 0.004719475 m^3^ fbm^-1^ for coastal harvest and 0.00492467 m^3^ fbm^-1^ for interior harvest. These factors were from the conversion rates documented in 1952–1955 annual reports and represent the historical scaling and milling practices [40]. I obtained wood density (oven-dry weight per unit of green-wood volume), by species from the regional literature [41,69]. Then I calculated an annual average wood density weighted by the harvest volume by species to convert the annual harvest into softwood and hardwood mass. I then multiplied the mass by 0.5 to get tonnes of C [70]. Softwoods accounted for 97–100% of the harvested volume. The weighted wood density for the softwood harvest ranged from 386 to 416 kg m^-3^. The annual hardwood wood density ranged from 338 (cottonwood only) to 443 kg m^-3^. The denser Douglas-fir dominate the initial logging industry in the province, however this changed in the 1970s to a greater proportion of spruce and pine species, resulting in a much flatter trend for C harvested than volume (Figure9).

I generated an ensemble of six sets of input data to represent the range of possible future harvests by first compiling the maximum sustainable harvest modelled in timber supply analyses [71]. BC regulators use these analyses to set a maximum annual allowable cut. Historically, the actual harvest has been as low as 46% of that maximum (in 1966 and 1967), recently dropping to 66% in 2009 [17]. Therefore, I used a simple random number generator to produce annual proportions from 61 to 100% and multiplied the proportions by the maximum harvest. The lower boundary is arbitrary and intended to help communicate the inherent uncertainty in predicting the future. Finally, I multiplied the volume by 403 kg m^-3^ – the average wood density from 1965 to 2010, and by 0.5 to get tonnes of C [70].

I input estimates of annual harvested C from 1965 to 2065 to the BC-HWPv1. This period includes most of the recorded harvest in BC (Figure9). Prior to 1965, there is little information on manufacturing primary products other than lumber and sparse data to verify model output. The IPCC Guidelines [8] recommend starting in 1900, but that date does not take into account national or regional circumstances such as the relatively low amount of harvesting in BC in the early part of the 20^th^ century.

### Building life-span data

A literature search on building life-spans identified only two published empirical datasets for the US and Canada. Winistorfer and others [49] used the housing census of the USA to estimate annual percent loss rates of 0.02 to 0.5% which would correspond to first-order decay half-lives of over 180 years. Athena [48] surveyed the age of demolished buildings in St. Paul Minnesota; the weighted average age of demolished wood buildings was 80 years. Their survey included residential and commercial buildings.

To add to the published empirical data, the BC Assessment provided datasets on the building stocks on January 1, 2000, and on July 31, 2011, and annual demolition permit information from 2000–2010. Of the over 17,000 demolition permits on record, I removed those properties with no change in the age of construction after the demolition permit was obtained (i.e. no demolition occurred), and those without a year of construction for the original building. The analysis showed that the demolition rate for buildings less than 40 years old is essentially zero (Table10). Older than 40, the rate varies from 0.2 to 0.4% per year which was consistent with half-lives of 158 to 376 years. However, there was only a short period of demolition data and a substantial amount of properties with unknown ages. The average age of buildings when demolished was 61 years for single family homes, 59 years for multi-family homes and commercial buildings, 40 years for moveable homes, and 45 years for recreational dwellings. But the average age reflects the distribution of buildings among different ages since the rate of construction has varied over time.

**Table 10:** Building stock construction period distribution in 2000 and demolition information from 2000–2010 for British Columbia

**Period of construction**	**Single-family house (%)**	**Multi-family dwelling (%)**	Moveable dwelling* (%)	**Recreational dwelling (%)**	Number of buildings in 2000**	**Demolitions from 2000–2010 (count)**	**Proportion of stock demolished over 10 years (%)**
<=1910	2	0.3	0	1	13,696	250	2
1911-1920	2	0.4	0	0	19,104	560	3
1921-1930	3	0.2	0	1	25,783	1,050	4
1931-1940	3	0.1	0	2	23,311	635	3
1941-1950	7	0.2	0	4	58,610	2,511	4
1951-1960	12	1	0.1	7	100,543	1,909	2
1961-1970	13	2	0.2	19	120,197	325	0
1971-1980	23	15	1	25	249,633	270	0
1981-1990	17	19	1	13	218,200	96	0
1991-2000	15	28	1	11	237,819	60	0
2001-2010						14	N/A
Unknown	4	33	97	17	228,355	3,021	
Total	101	100	100	100	1,295,251	7,680	1

Data were from the British Columbia Assessment demolition permit and property assessment database.

* Manufactured homes.

** Residential, commercial and institutional.

The variable rate of construction can be captured using building permits with the assumption that all construction has a permit and all permits are in the dataset. Statistics Canada [72] provided the number of building permits issued by BC municipalities over time (Table11). While this is a longer dataset than the one for demolitions, there are some limitations, since the municipalities responding to the Statistics Canada survey have changed over time. For the 2001–2010 decade, the demolition and building permit datasets overlap. In 2001–2010, municipalities issued 291,814 building permits for dwellings, but there were 261,226 dwellings in the database of existing properties. Since only 14 houses were demolished (Table10), then about 30,588 or 10.5% of the building permits must not have resulted in a dwelling actually being built. Therefore, I reduced the number of all building permits by 10.5%. I then compared the adjusted building permit data to the remaining number of dwellings in 2011. Based on this comparison, loss rates range from 0.0 to 0.6% per year, corresponding to first-order decay half lives from 90 to 980 years for housing in BC. 

**Table 11:** Housing stock in 2011, and building permits from 1961–2010 for British Columbia by period of construction

**Period of construction**	**Adjusted number of building permits per decade**	**Number of dwellings in 2011**	**2011 dwelling stock as proportion of building permits (%)**	**Loss of dwelling stock based on building permit survey (% per decade)**
<=1910		14,749		
1911-1920		19,743		
1921-1930		24,100		
1931-1940		21,929		
1941-1950		53,248		
1951-1960		94,847		
1961-1970	179,190	121,666	68	6
1971-1980	290,927	257,696	89	3
1981-1990	236,003	231,815	98	1
1991-2000	260,817	275,815	106	0
2001-2010	261,240	261,226	100	0
Unknown		76,792		

Data were from the British Columbia Assessment property assessment database and Statistics Canada [72].

I checked for similar building stock and permit information nationally. The Canadian Census surveys ask a 20% sample of households for the period of construction of their residence [73,74]. The 10-year decline in housing stock (1996 to 2006) was apparently within sampling error until dwellings were more than 60 years old (data not shown). For the oldest homes (built before 1946), the annual loss rate was about 0.7%, which corresponds to half-lives 89 to 160 years. Most dwellings (58%) were built since 1971, however 6-7% of the housing stock was older than 85 years in 2006 [74]. Only moveable dwellings had a substantially different distribution with 86% begin built since 1971. Of course, the distribution of housing stock among different periods of construction depends on the building rate as well as its life-span. Unfortunately, the number of building permits issued by municipalities across all of Canada did not provide useable data on the number of dwellings built [75]. This may have been because the survey area has changed over time, or because it did not include smaller municipalities and rural areas which are represented in the census data of housing stock (data not shown).

Less information was available specifically for commercial buildings. A survey of public schools in 1999 for the USA found that schools originally built in 1901 were still in use in and almost half of the schools were over 40 years old [76] (Table12). Two-thirds of the schools had undergone major renovations. The USA Department of Energy building stock was on average over 30 years old in 2002 [77]. 

**Table 12:** Distribution of US schools amoung different periods of construction

**Period of construction**	**Age class**	**Proportion of schools surveyed (%)**
1901-1920	80	3
1921-1930	70	7
1931-1940	60	5
1941-1950	50	6
1951-1960	40	26
1961-1970	30	24
1971-1980	20	13
1981-1990	10	8
1991-2000	0	9

Data were from Lewis and others [76].

### Calculation of GHG emissions

Calculation of GHG emissions used the same approach and global warming factors as the Canadian inventory [3]. To estimate the GHG emissions, I took the annual stock change in ECO_2_ and multiplied by 44/12 to convert from tonnes of C to CO_2_. To this, I added the annual stock change in ECH_4_ after multiplying by 16/12 to convert the molar mass and by 21 to account for the global warming potential of methane. When the emissions were caused by combustion of wood or paper I additionally estimated the amount of N_2_O to be the equivalent of 0.00008% of C emitted multiplied by 44/12 to convert the molar mass and by 310 to account for the global warming potential.

### Verification, uncertainty and sensitivity methods

Commodity production statistics provide an opportunity to verify some of the model parameters. Sawn lumber production data [39] had to be converted from cubic metres of rough green lumber to dry planed lumber. I followed Briggs [45] in converting plywood and veneer statistics into cubic meters. To convert from cubic meters to biomass I obtained wood density (oven-dry mass per unit of dry volume) by species from Gonzales [69] and Nielson and others [41]. I created a weighted wood density from the 5-year harvest volume by species to convert the annual products into mass which I then multiplied by 0.5 to get tonnes of C [70]. The weighted dry wood density ranged from 433 to 445 kg m^-3^ between 1965 and 2010. I converted the panel statistics from cubic meters to tonnes of biomass using densities of 500 kg m^-3^. Paper production and pulp export statistics were available as air dry tonnes [39]. To convert to C, I multiplied mechanical pulp and paper by 0.5 and chemical pulp and paper by 0.4 [42].

One source of uncertainty is the export of logs, chips, sawdust, shavings and other waste wood from BC to other countries because the receiving manufacturer may have a different product recovery rate or waste handling procedures. Exports have ranged from about 0.5 to 1.5 Mt C per year since 1988 (Figure8) [39]. Another source of uncertainty comes from the range of potentially valid parameter values. In each table of parameters, the minimum and maximum values may be unlikely, but are still possible.

To assess the effect of the range of different parameter values, I constructed simulations with the combinations of parameters which would maximize or minimize the GHG emissions. The half-life parameters in the BC-HWPv1 are generally higher than the defaults published by the IPCC [8]. To assess the error the model would have if it used the simpler structure of in-use pools and half-lives recommended by the IPCC, I ran a version of the simulation with paper set to a half-life of 2 years (instead of 2.5) and all wood products to a half-life of 30 years (instead of a range from 2 to 90 years).

## Competing interests

The author declare that they have no competing interests.
